# Internal Jugular Vein Cross-Sectional Area and Cerebrospinal Fluid Pulsatility in the Aqueduct of Sylvius: A Comparative Study between Healthy Subjects and Multiple Sclerosis Patients

**DOI:** 10.1371/journal.pone.0153960

**Published:** 2016-05-02

**Authors:** Clive B. Beggs, Christopher Magnano, Pavel Belov, Jacqueline Krawiecki, Deepa P. Ramasamy, Jesper Hagemeier, Robert Zivadinov

**Affiliations:** 1 Buffalo Neuroimaging Analysis Center, Department of Neurology, School of Medicine and Biomedical Sciences, University at Buffalo, Buffalo, New York, United States of America; 2 MRI Clinical Translational Research Center, School of Medicine and Biomedical Sciences, University at Buffalo, Buffalo, New York, United States of America; 3 Research Institute for Sport, Physical Activity and Leisure, Carnegie Faculty, Leeds Beckett University, Leeds, West Yorkshire, United Kingdom; Instituto Cajal-CSIC, SPAIN

## Abstract

**Objectives:**

Constricted cerebral venous outflow has been linked with increased cerebrospinal fluid (CSF) pulsatility in the aqueduct of Sylvius in multiple sclerosis (MS) patients and healthy individuals. This study investigates the relationship between CSF pulsatility and internal jugular vein (IJV) cross-sectional area (CSA) in these two groups, something previously unknown.

**Methods:**

65 relapsing-remitting MS patients (50.8% female; mean age = 43.8 years) and 74 healthy controls (HCs) (54.1% female; mean age = 43.9 years) were investigated. CSF flow quantification was performed on cine phase-contrast MRI, while IJV-CSA was calculated using magnetic resonance venography. Statistical analysis involved correlation, and partial least squares correlation analysis (PLSCA).

**Results:**

PLSCA revealed a significant difference (p<0.001; effect size = 1.072) between MS patients and HCs in the positive relationship between CSF pulsatility and IJV-CSA at C5-T1, something not detected at C2-C4. Controlling for age and cardiovascular risk factors, statistical trends were identified in HCs between: increased net positive CSF flow (NPF) and increased IJV-CSA at C5-C6 (left: r = 0.374, p = 0.016; right: r = 0.364, p = 0.019) and C4 (left: r = 0.361, p = 0.020); and increased net negative CSF flow and increased left IJV-CSA at C5-C6 (r = -0.348, p = 0.026) and C4 (r = -0.324, p = 0.039), whereas in MS patients a trend was only identified between increased NPF and increased left IJV-CSA at C5-C6 (r = 0.351, p = 0.021). Overall, correlations were weaker in MS patients (p = 0.015).

**Conclusions:**

In healthy adults, increased CSF pulsatility is associated with increased IJV-CSA in the lower cervix (independent of age and cardiovascular risk factors), suggesting a biomechanical link between the two. This relationship is altered in MS patients.

## Introduction

Many studies have linked constriction of the internal jugular veins (IJVs) with retention of venous blood in the cranium [1] and increased intracranial pressure (ICP) [2–5]. Constriction of the IJVs has also been shown to stiffen the brain parenchyma [6] and an increase in the amplitude of the cerebrospinal fluid (CSF) pulse in the aqueduct of Sylvius (AoS) [6, 7] in healthy individuals. Collectively, these findings indicate that cervical venous drainage plays an important role in regulating both ICP [8] and intracranial fluid dynamics [9, 10]. They also suggest that anomalies in the cervical venous system may have an adverse impact on the physiology of the intracranial space. Indeed, a number of studies have linked constricted cerebral venous outflow with neurodegenerative disorders, such as multiple sclerosis (MS) [11–13], Parkinson's disease [14], Meniere’s disease [15, 16] and Alzheimer’s disease [17, 18].

While the link between constricted cerebral venous outflow and neurological disease is poorly understood, a number of studies have linked MS with increased CSF pulsatility in the AoS [19–21], raising questions about whether or not the phenomenon might be associated with a venous abnormality. The hydraulic resistance of the cerebral venous drainage pathways has been found to increase by 63% in MS patients [12, 22], reportedly due to the presence of stenotic vessels [11] and collateral rerouting of the venous blood flow back to the heart [23]. However, it is not known whether aqueductal CSF pulsatility is biomechanically linked to the venous system in MS patients, as it appears to be in healthy individuals [6, 7], or whether other factors such as ventricular enlargement [24] and parenchymal atrophy [25] influence its behaviour. In order to establish whether or not MS patients behave differently from healthy individuals regarding this issue, we undertook a study involving 65 relapsing-remitting MS patients and 74 healthy controls, to investigate the relationship between IJV cross-sectional area (CSA) and the dynamics of the aqueductal CSF pulse in both groups.

## Materials and Methods

### Patient population

This study utilized data from an ongoing prospective study of cardiovascular, environmental and genetic risk factors in neurologic diseases and in healthy individuals [26, 27]. 65 relapsing-remitting MS patients (32 males and 33 females; mean age = 43.8 years) and 74 non-familial healthy controls (34 males and 40 females; mean age = 43.9 years) who underwent magnetic resonance imaging (MRI) scan with cine phase contrast (PC) imaging and magnetic resonance venography (MRV) were included. The individuals needed to qualify on a health screening questionnaire containing information about medical history (illnesses, surgeries, medications, etc.) and meet the health screen requirements for MRI on physical examination, as previously described [27–29]. Exclusion criteria were: pre-existing medical conditions known to be associated with brain pathology (e.g. cerebrovascular disease, positive history of alcohol abuse, etc.), history of cerebral congenital vascular malformations, or pregnancy. Relevant information relating to cardiovascular risk factors [body mass index (BMI), hypertension, diabetes, cardiovascular disease and smoking] was collected. In addition, the clinical symptoms of the MS patients were assessed, with each patients being assigned expanded disability status scale (EDSS) [30] and multiple sclerosis severity score (MSSS) [31] scores.

All participants underwent clinical and MRI examinations. The study was approved by the University of Buffalo Institutional Review Board and written informed consent was obtained from all subjects.

### MRI acquisition

All subjects were examined on a 3 Tesla GE Signa Excite HD 12.0 Twin Speed scanner (General Electric, Milwaukee, WI). All sequences were run on an 8-channel head and neck neurovascular coil. All analyses were performed in a blinded manner.

CSF flow quantification was performed using a single slice cine phase-contrast velocity-encoded pulse-gated gradient echo sequence (cine PC) with an echo time (TE)/repetition time (TR)/flip angle (FA) of 7.9 ms/40 ms/20°, a slice thickness of 4 mm, a velocity encoding of 20 cm/s and 32 phases acquired corresponding to the cardiac cycle. The cine PC sequence was acquired with a 256x256 matrix over a 10.0 cm field of view (FOV) for a resolution of 0.39 x 0.39 x 4 mm^3^ with the AoS prescribed centrally, such that the wrap around artifact was present in the edges of the FOV, but did not overlap with the desired region of interest (ROI). A sagittal T2-weighted fast SE sequence was also acquired as a localizer for the cine PC prescription, as previously described, with the cine PC sequence prescribed as an oblique axial slice through the AoS [20]. All subjects underwent the MRI exam during the same time of day (in the afternoon hours) to control for circadian variation.

A 2-dimensional MRV sequence was acquired for all internal jugular vein (IJV) cross-sectional area (CSA) measurements. The MRV was acquired with 150, 1.5mm-thick slices using a 320x192 matrix (frequency x phase) over a 22.0 cm FOV and a phase FOV of 75% for a resolution of 0.69 x 1.15 x 1.5 mm^3^. Additional imaging parameters included TE/TR/FA 4.3 ms/14 ms/70°, and a bandwidth (BW) of 31.25 kHz. MRV was acquired in a true (non-obliqued) axial orientation with one average, and no parallel imaging techniques were employed.

### Image analyses

As previously described by Magnano [20], CSF Flow metrics were assessed using a combination of GE ReportCard software (version 3.6; General Electric, GE, Milwaukee, WI) and a semi-automated in-house semi-automated minimum area of contour change (MACC) program [32]. Briefly, ReportCard was used to calculate average velocity over the AoS at all measured 32 phases of the cardiac cycle. ACC takes a seed point and, on all 32 phases, selects a surrounding iso-contour curve, which marks the steepest overall gradient of image intensity values. In this case, on the magnitude images, the AoS is bright due to flow, whereas the background tissue with no flow is dark, and MACC can accurately outline the AoS ROI with sub-voxel accuracy which also greatly improves inter-operator variability, as previously published [32].

CSF flow direction was calculated based on slice prescription such that flow through the AoS out of the slice (during diastole, towards the third ventricle) was given as positive, whereas flow into the slice (during systole, towards the fourth ventricle) was negative, as described previously [20]. Using these raw velocity and flow values at each phase of the cardiac cycle, the summation of only the positive or negative flows resulted in our net positive and net negative flows (NPF and NNF), respectively. The net flow (NF) was calculated as the overall sum of flows over all 32 phases (which can also be calculated as (NF = NNF + NPF). The peak positive and negative velocities (PPV, PNV) were assessed as the maximum positive or minimum negative velocity out of all 32 phases. It is important to note that the peak velocities are the peak average velocity of the entire AoS ROI and not a peak single voxel which could be an outlier. CSF flow measures are presented in microliters per beat (μL/beat, 1μL = 1mm^3^), while CSF velocity measures are presented in cm/s.

### Image analyses of MRV

IJV assessment was performed using CSA ROI analysis on the 2D MRV with the Java Image Manipulation Tool (JIM) version 5.0 (http://www.xinapse.com) at specific cervical locations as previously described [33]. Briefly, the sequence was viewed orthogonally to assess which slices corresponded to the desired anatomical coverage, namely C2-C3, C4, C5-C6, and C7-T1. Within each of these locations, the operator determined the slice on which the IJV came to a minimum, and then used the ROI Toolkit to select the right and left IJVs. Most commonly, this was accomplished using the Contour ROI tool, using the automated Preview Contours tool to best select its edges. When necessary, the operator manually adjusted the ROI boundary.

### Statistical analysis

The demographic, clinical, and MRI (derived CSF and IJV-CSA) measures are listed in Table 1. Statistical analysis was undertaken using in-house algorithms written in Matlab (Mathworks, Natick, Mass) and R (open source statistical software). Univariate statistical analysis was performed using Student’s t-test (two-tailed) and the Chi-square test. Due to multiple comparisons, only nominal values of p<0.01 for two-tailed tests were considered statistically significant.

**Table 1:** Descriptive statistics of the demographic, cardiovascular risk factor, MRI cerebrospinal fluid and magnetic resonance venography data.

	Healthy	RR MS	Significance
Variable	Subjects	Patients	p value
	(n = 74)	(n = 65)	
Age (years); mean (SD)	43.9 (18.3)	43.8 (10.2)	0.969
Female sex; n (%)	40 (54.1)	33 (50.8)	0.699
BMI (kg/m^2^); mean (SD)	26.11 (5.85)	25.95 (5.41)	0.871
Current smokers; n (%)	7.0 (9.5)	5.0 (7.7)	0.711
Hypertension; n (%)	5.0 (6.8)	3.0 (4.6)	0.589
Diabetes mellitus type 1; n (%)	0.0 (0.0)	0.0 (0.0)	na
Cardiovascular disease; n (%)	5.0 (6.8)	8.0 (12.3)	0.262
NF (μL/beat); mean (SD)	-3.70 (7.15)	-3.97 (11.32)	0.871
NNF (μL / beat); mean (SD)	-29.14 (15.87)	-36.16 (21.75)	0.034*
NPF (μL / beat); mean (SD)	25.44 (14.83)	32.19 (18.53)	0.020*
PPV (cm/s); mean (SD)	6.48 (2.59)	7.28 (2.87)	0.088
PNV (cm/s); mean (SD)	-8.03 (2.71)	-9.38 (5.17)	0.061
C7-T1 RIJV CSA (mm2); mean (SD)	73.69 (54.75)	62.10 (48.44)	0.188
C7-T1 LIJV CSA (mm2); mean (SD)	47.38 (34.60)	41.93 (32.05)	0.337
C5-C6 RIJV CSA (mm2); mean (SD)	59.43 (42.58)	51.21 (38.82)	0.236
C5-C6 LIJV CSA (mm2); mean (SD)	43.27 (28.88)	38.65 (30.42)	0.362
C4 RIJV CSA (mm2); mean (SD)	55.34 (32.07)	50.16 (24.55)	0.285
C4 LIJV CSA (mm2); mean (SD)	37.74 (22.57)	39.96 (23.02)	0.570
C2-C3 right collateral CSA (mm2); mean (SD)	10.36 (5.62)	10.03 (6.11)	0.747
C2-C3 RIJV CSA (mm2); mean (SD)	42.72 (28.11)	39.29 (21.62)	0.421
C2-C3 LIJV CSA (mm2); mean (SD)	27.08 (19.62)	27.08 (20.36)	1.000
C2-C3 left collateral CSA (mm2); mean (SD)	10.74 (5.77)	9.80 (5.89)	0.345

RR MS, relapsing-remitting multiple sclerosis, BMI, body mass index; NF, net flow (i.e. NNF minus NPF); NNF, net negative flow; NPF, net positive flow; PPV, peak positive velocity; PNV, peak negative velocity; RIJV, right internal jugular vein; LIJV, left internal jugular vein; CSA, cross sectional area; na, not applicable.

* p values less than 0.05 considered trends

Partial least squares correlation analysis (PLSCA) was undertaken to establish the strength of the relationships between the various component sub-groups within the data [34]. This involved performing PLSCA on the observed data to determine the inertia (sum of the singular values) of the covariance matrix of the groups of variables under consideration, as previously described [34–36]. Because singular values are proportional to the magnitude of any effect [35], the higher the value of the inertia observed, the greater the amount of shared information between the chosen sub-groups. Having established the singular value inertia of the measured data, a permutation test involving 100,000 random permutations with replacement was performed to establish the sample distribution of the possible inertias and the likelihood (the odds) of the observed relationship occurring by chance [36].

PLSCA was performed separately on the data collected from the MS patients and the healthy controls, with CSF NPF and NNF being compared with IJV-CSA in the lower (C5-T1) and upper (C2-C4) neck. The relationship between the CSF variables and the left and right IJV-CSAs was also investigated. The differences between the odds calculated for the respective groups were tested using a Chi-square test, with the corresponding effect sizes calculated using Cramer’s V test. Pearson partial correlation analysis was also performed to quantify the relationships between the MRI (CSF pulse) and MRV (IJV-CSA) variables after controlling for age, BMI, hypertension, smoking and cardiovascular disease. A two-tailed sign test (significance set at p<0.05) was used to assess changes in the r-values between the two groups, with positive values assigned to correlations that strengthened and negative values to correlations that weakened.

The relationship between EDSS, MSSS and the MRV (IJV-CSA) variables in the MS patients was evaluated using Spearman partial correlation analysis, with age, BMI, hypertension, smoking and cardiovascular disease as covariates.

## Results

### Demographic and clinical characteristics

Table 1 shows the demographic, clinical, CSF MRI and MRV characteristics. The average age of the healthy controls was 43.9 years (SD = 18.3 years), with females comprising 53% of this cohort, whereas the average age of the MS patients was 43.8 years (SD = 10.2 years), with females comprising 51%. In the MS group, average disease duration was 11.6 years (SD = 8.1 years), with the mean EDSS and MSSS scores being 2.4 (SD = 1.3) and 2.9 (SD = 2.0), respectively (Table 2).

**Table 2:** Descriptive statistics of the clinical characteristics of the relapsing-remitting multiple sclerosis patients.

	RR MS
Variable	Patients
	(n = 65)
Age (years); mean (SD)	43.8 (10.2)
Age of onset (years); mean (SD)	32.1 (8.5)
Disease duration (years); mean (SD)	11.6 (8.1)
EDSS score; mean (SD)	2.4 (1.3)
MSSS; mean (SD)	2.9 (2.0)

RR MS, relapsing-remitting multiple sclerosis; EDSS, expanded disability status scale; MSSS, multiple sclerosis severity scores.

**Fig 1:**
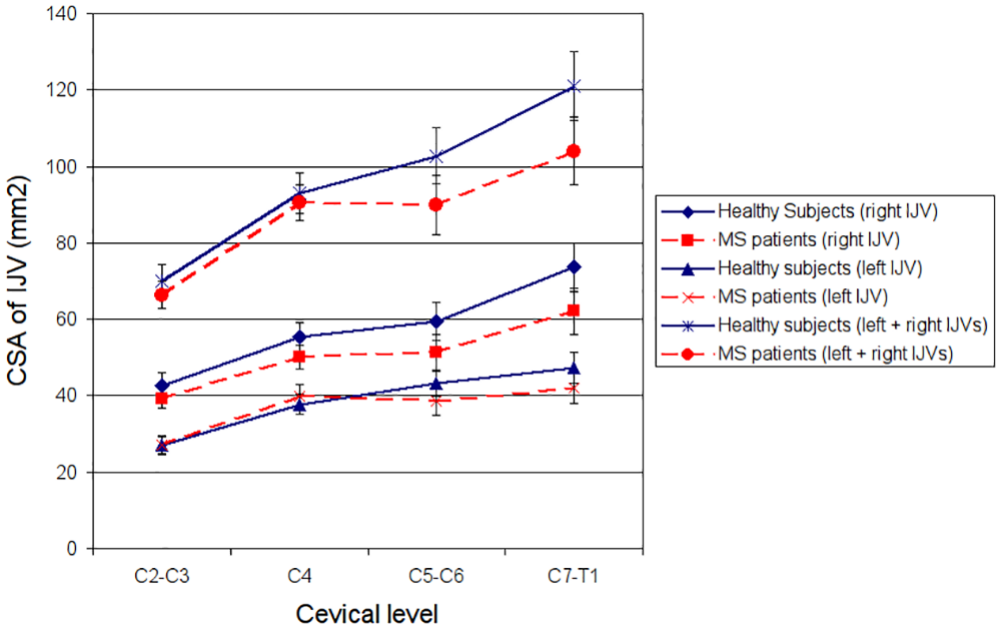
Cross sectional areas (CSAs) of respective left and right internal jugular veins (IJVs) in the MS patients and healthy subjects. (Error bars represent one standard error of the mean.)

Analysis of the clinical characteristics revealed no statistical difference between the healthy controls and the MS patients (Table 1). Both groups exhibited a net aqueductal CSF flow of similar magnitude in the caudal direction. However, in the MS patients there was a statistical trend towards increased NNF (p = 0.034) and NPF (p = 0.020) compared with the healthy controls. At the lower cervical levels (C5-T1), the left and right hand IJV-CSAs were smaller in the MS patients compared with the healthy controls (Fig 1), but this did not reach significance. In the upper cervix (C2-C4) there was little difference in IJV-CSA between the two cohorts.

### Partial least squares correlation analysis

**Fig 2:**
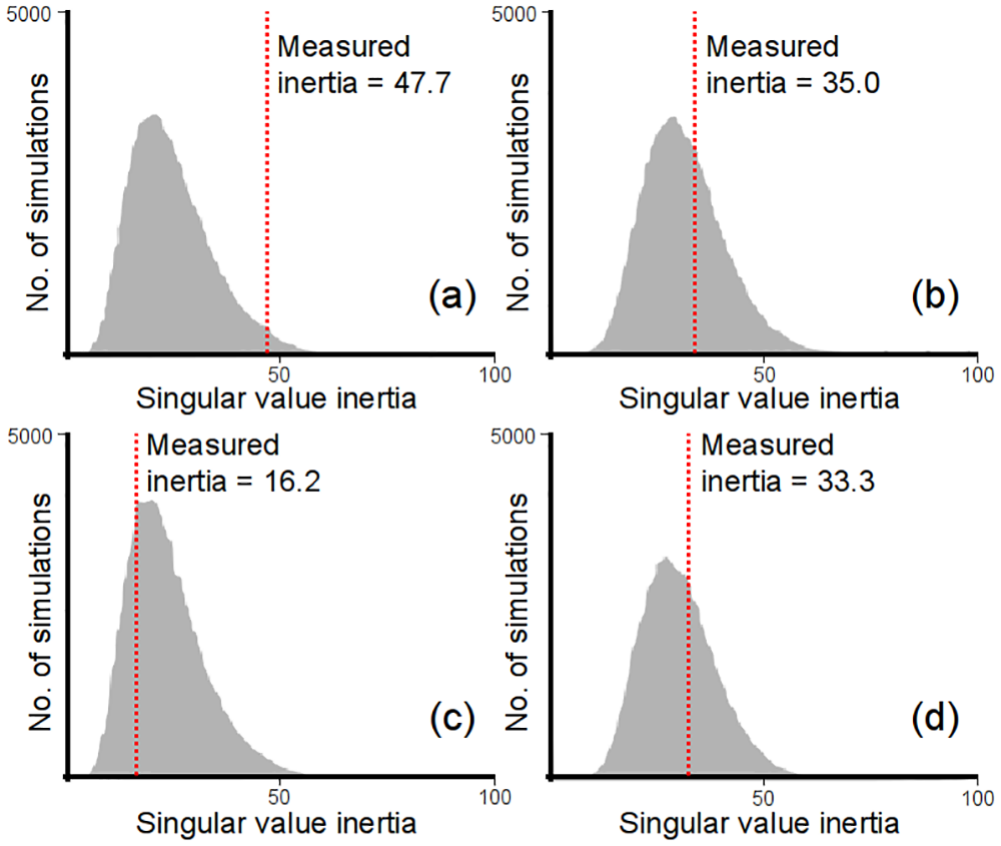
Results of the partial least squares correlation analysis (100000 simulations) comparing the cerebrospinal fluid variables with IJV cross-sectional areas for: (a) healthy controls, lower neck (C5-T1); (b) healthy controls, upper neck (C2-C4); (c) MS patients, lower neck (C5-T1); (d) MS patients, upper neck (C2-C4).

The PLSCA results are presented in Fig 2 and Table 3. Fig 2 shows the sample distributions of the possible inertia values for the various analyses undertaken. The further the measured inertia value is to the right of the sample distribution, the stronger the relationship between the two groups of variables being compared [35]. The PLSCA results for the lower cervix (C5-T1) (Fig 2a and 2c) revealed the measured inertia (47.65; odds = 25:1000) in the healthy subjects to be much greater than the corresponding value in the MS patients (16.21; odds = 767:1000), with the difference between the odds being highly significant (p<0.001; effect size = 1.072). By comparison, for the upper cervix (C2-C4) (Fig 2b and 2c) the measured inertia (33.34; odds = 361:1000) in the MS patients was very similar to that for the healthy controls (35.03; odds = 347:1000). In both cohorts the within group difference between the odds for the upper and lower cervical veins was significant (p<0.001; effect size > 0.570), although the direction of this difference was opposite in the MS cohort compared with the healthy group.

**Table 3:** Results of the partial least squares correlation analysis comparing the cerebrospinal fluid variables with internal jugular vein cross-sectional areas for the lower neck (C5-T1) and upper neck (C2-C4), in both the MS patients and the healthy controls (based on 100000 simulations).

Subjects	Group 1 variables	Group 2 variables	No. subjects included	Measured Inertia	Odds	Significance p value	Effect size (Cramer’s V)
Healthy subjects	NNF, NPF	C7-T1 RIJV CSA, C7-T1 LIJV CSA, C5-C6 RIJV CSA, C5-C6 LIJV CSA	71	47.65	25:1000	<0.001**^ <0.001**^^	1.072^ 0.585^^
Healthy subjects	NNF, NPF	C4 RIJV CSA, C4 LIJV CSA, C2-C3 right collateral CSA, C2-C3 RIJV-CSA, C2-C3 LIJV CSA, C2-C3 left collateral CSA	71	35.03	347:1000	0.513^ <0.001**^^	0.021^ 0.585^^
RR MS patients	NNF, NPF	C7-T1 RIJV CSA, C7-T1 LIJV CSA, C5-C6 RIJV CSA, C5-C6 LIJV CSA	63	16.21	767:1000	<0.001**^ <0.001**^^	1.072^ 0.579^^
RR MS patients	NNF, NPF	C4 RIJV CSA, C4 LIJV CSA, C2-C3 right collateral CSA, C2-C3 RIJV CSA, C2-C3 LIJV CSA, C2-C3 left collateral CSA	63	33.34	361:1000	0.513^ <0.001**^^	0.021^ 0.579^^

RR MS, relapsing-remitting multiple sclerosis, NNF, net negative flow; NPF, net positive flow; PPV, peak positive velocity; PNV, peak negative velocity; RIJV, right internal jugular vein; LIJV, left internal jugular vein; CSA, cross sectional area.

^ Comparison of the between-group odds for the healthy subjects and RR MS patients

^^ Comparison of the within-group odds for the respective healthy subject and RR MS patient groups

* p values less than 0.05 considered trends using Chi-square test

** p values less than 0.01 considered significant using Chi-square test

### Correlation analysis

After controlling for age, BMI, hypertension, smoking and cardiovascular disease, the correlation analysis (Tables 4 and 5) revealed statistically positive trends in the healthy controls between: NPF and left IJV-CSA at C5-C6 (r = 0.374, p = 0.016) and C4 (r = 0.361, p = 0.020); NPF and right IJV-CSA at C5-C6 (r = 0.364, p = 0.019); and PPV and left IJV-CSA at C7-T1 (r = 0.336, p = 0.032) and C4 (r = 0.351, p = 0.024), while negative trends were observed between NNF and left IJV-CSA at: C5-C6 (r = -0.348, p = 0.026) and C4 (r = -0.324, p = 0.039). By comparison, in the MS patients a positive trend was only identified between NPF and right IJV-CSA at C5-C6 (r = 0.351, p = 0.021). Correlations between NPF and left IJV-CSA were much weaker (i.e. r<0.1) in the MS cohort compared with those in the healthy subjects. Overall the correlations were generally weaker in the MS patients compared with the healthy controls, with the magnitude of the r-values reducing or changing direction in 34 out of the 50 correlations (p = 0.015).

**Table 4:** Results of the Pearson partial correlation analysis comparing the cerebrospinal fluid and internal jugular vein cross-sectional area variables, when controlling for age, BMI, hypertension, smoking & cardiovascular disease for the healthy controls (n = 74).

	NF, r (p value)	NNF, r (p value)	NPF, r (p value)	PPV, r (p value)	PNV, r (p value)
C7-T1 RIJV CSA	0.075 (0.639)	-0.235 (0.139)	0.299 (0.058)	0.229 (0.149)	0.007 (0.963)
C7-T1 LIJV CSA	-0.068 (0.672)	-0.294 (0.062)	0.296 (0.060)	0.336 (0.032)*	-0.152 (0.344)
C5-C6 RIJV CSA	0.154 (0.338)	-0.259 (0.102)	0.364 (0.019)*	0.307 (0.051)	0.03 (0.851)
C5-C6 LIJV CSA	-0.032 (0.841)	-0.348 (0.026)*	0.374 (0.016)*	0.24 (0.131)	-0.178 (0.266)
C4 RIJV CSA	0.008 (0.959)	-0.180 (0.259)	0.206 (0.197)	0.276 (0.080)	0.000 (0.999)
C4 LIJV CSA	-0.003 (0.985)	-0.324 (0.039)*	0.361 (0.020)*	0.351 (0.024)*	-0.146 (0.362)
C2-C3 right collateral CSA	0.151 (0.347)	0.014 (0.930)	0.057 (0.726)	-0.054 (0.735)	0.182 (0.256)
C2-C3 RIJV CSA	0.101 (0.530)	-0.044 (0.783)	0.098 (0.542)	0.079 (0.624)	0.060 (0.708)
C2-C3 LIJV CSA	0.077 (0.634)	-0.163 (0.309)	0.219 (0.169)	0.28 (0.076)	-0.070 (0.664)
C2-C3 left collateral CSA	0.056 (0.728)	-0.052 (0.748)	0.085 (0.599)	0.075 (0.643)	-0.081 (0.614)

NF, net flow; NNF, net negative flow; NPF, net positive flow; PPV, peak positive velocity; PNV, peak negative velocity; RIJV, right internal jugular vein; LIJV, left internal jugular vein; CSA, cross sectional area.

* p values less than 0.05 considered statistical trends

**Table 5:** Results of the Pearson partial correlation analysis comparing the cerebrospinal fluid and internal jugular vein cross-sectional area variables, when controlling for age, BMI, hypertension, smoking & cardiovascular disease for the relapsing-remitting MS patients (n = 65).

	NF, r (p value)	NNF, r (p value)	NPF, r (p value)	PPV, r (p value)	PNV, r (p value)
C7-T1 RIJV CSA	0.143 (0.361)	-0.139 (0.375)	0.256 (0.098)	0.108 (0.489)	0.044 (0.779)
C7-T1 LIJV CSA	-0.12 (0.444)	-0.133 (0.393)	0.079 (0.613)	-0.042 (0.789)	-0.05 (0.748)
C5-C6 RIJV CSA	0.109 (0.485)	-0.238 (0.124)	0.351 (0.021)*	0.067 (0.668)	0.141 (0.366)
C5-C6 LIJV CSA	-0.150 (0.335)	-0.138 (0.377)	0.065 (0.678)	-0.153 (0.327)	0.043 (0.782)
C4 RIJV CSA	0.204 (0.190)	-0.143 (0.361)	0.300 (0.051)	0.125 (0.424)	0.187 (0.229)
C4 LIJV CSA	-0.226 (0.145)	-0.201 (0.196)	0.090 (0.566)	0.005 (0.974)	0.000 (0.999)
C2-C3 right collateral CSA	-0.124 (0.428)	-0.192 (0.218)	0.145 (0.352)	-0.083 (0.597)	0.065 (0.679)
C2-C3 RIJV CSA	0.169 (0.277)	-0.151 (0.332)	0.288 (0.061)	0.078 (0.620)	0.179 (0.251)
C2-C3 LIJV CSA	-0.142 (0.362)	-0.071 (0.652)	-0.009 (0.954)	-0.010 (0.948)	0.039 (0.802)
C2-C3 left collateral CSA	-0.157 (0.314)	-0.271 (0.079)	0.217 (0.162)	-0.071 (0.649)	0.149 (0.340)

NF, net flow; NNF, net negative flow; NPF, net positive flow; PPV, peak positive velocity; PNV, peak negative velocity; RIJV, right internal jugular vein; LIJV, left internal jugular vein; CSA, cross sectional area.

* p values less than 0.05 considered statistical trends

In the MS patients, the correlations between the measures of disability and the MRV (IJV-CSA) variables were generally weak (EDSS: p<0.147; MSSS: p<0.238), with none reaching significance.

## Discussion

The principal finding of the study, highlighted by the PLSCA (Fig 2), is that at the lower cervical levels (C5-T1), the MS patients exhibited differences in the relationship between IJV-CSA and the aqueductal CSF variables (NNF and NPF) compared with the healthy subjects, something that was not observed at the upper cervical levels (C2-C4). In the upper neck (Fig 2b and 2d), the strength of this relationship (quantified by the magnitude of the singular value inertia of the covariance matrix of the measured data) was very similar in both groups, whereas for levels C5-T1 (Fig 2a and 2c) the inertia was much lower in the MS patients compared with the healthy subjects. Because inertia is a measure of the strength of any relationship that exist, this indicates that with regard to the upper neck the two groups behaved in a very similar manner, whereas at the lower cervical levels the biomechanical relationship between the CSF pulse and IJV-CSA was weaker in the MS cohort. This suggests that the difference between the groups is associated with changes in the IJVs at lower cervical levels in the MS patients. This is also supported by the results in Fig 1, which reveal the total IJV-CSA (sum of the right and left IJVs) to be smaller in the MS patients at levels C5-T1, despite being almost identical in both cohorts at levels C2-C4.

In keeping with other researchers [8, 37], we found the right IJV to be dominant and substantially larger than that of the left IJV in both the healthy controls and MS patients. However, it is noticeable from Fig 1 that, for both the left and right IJVs, there was little or no increase in CSA between levels C4 and C5-C6 in the MS patients, whereas in the healthy subjects the IJV-CSA steadily enlarged as the veins descended, reflecting the increase in venous blood flow that normally occurs towards the thorax [2, 38]. This difference between the groups may indicate the presence of collateral venous pathways [23] rerouting blood back to the heart in the MS patients, as postulated by Zamboni et al [11], or alternatively, it may be indicative of vein wall compression by the sternocleidomastoid muscle, just as Farina et al observed in MS patients [38]. While it is not possible to draw any firm conclusions about this from the data, the narrower IJVs in the lower neck of the MS patients may be indicative of increased hydraulic resistance in the cervical veins transporting blood back to the heart. The hydraulic resistance of the cerebral venous drainage pathways is known to increase in MS patients diagnosed with constricted venous outflow [22]. Furthermore, it is known that constriction of the IJVs increases both cerebral venous pressure and ICP due to increased resistance of the cerebral venous drainage pathways [3], something that has been shown to increase cerebral venous pressure and stiffness of the brain parenchyma, causing the amplitude of the aqueductal CSF pulse to increase [6]. If this process is at work in the MS patients, then we would expect to see a reduction in the IJV-CSA and an increase in the magnitude of NNF and NPF compared with the controls, which is exactly what was observed in this study (Table 1), in line with previous findings [20, 21].

Although the clinical implications of narrow IJVs are poorly understood, an association between reduced IJV size and increased EDSS score has been reported in MS patients [39, 40]. However, after controlling for age, BMI and cardiovascular risk factors, we observed no significant correlations between IJV-CSA and either EDSS score or MSSS score. The reasons for this are unclear and may be due to the fact that our study only included RR MS patients with a mean EDSS score of 2.4. Severe narrowing of the IJVs, characterised by chronic cerebrospinal venous insufficiency (CCSVI), is thought to be associated more with secondary progressive MS patients [27] and those with EDSS scores greater or equal to 6 [39]. However, our patient cohort only included three subjects with an EDSS score greater or equal to 6, something that may, in part, explain the lack of any significant relationship between EDSS and the MRV (IJV-CSA) variables.

BMI has been shown to be positively correlated with IJV-CSA in the lower neck of both MS patients and healthy individuals [33]. Likewise, IJV-CSA is known to increase with age [41]. When we controlled for both these covariates, as well as other cardiovascular risk factors (Tables 4 and 5), we found differences between the groups, with a general weakening of the correlations between the CSF and IJV-CSA variables in the MS patients compared with the healthy controls. In particular, the relationship between NPF and left IJV-CSA differed between the two groups, with a moderate positive effect size (r>0.29) observed in the healthy subjects at levels C4-T1, which was absent in the MS patients. By comparison, little difference was observed between the groups in the relationship between NPF and right IJV-CSA for the lower cervical levels. This implies that the principle differences between the MS patients and the healthy controls relate to changes associated with the left IJV in the mid-to-lower neck. Given that the correlations in Tables 4 and 5 relate to a single aqueductal pulse, if the characteristics of the IJVs on the left and right hand sides were evenly matched, then one would expect the respective r-values associated with NNF, NPF and IJV-CSA to be roughly similar for both sides of the neck, which is broadly what we observed for the mid-to-lower neck in the healthy cohort. If however, changes occurred on the left hand side in a substantial fraction of the subjects, say due to the rerouting of blood through collateral veins, then one would observe a general weakening of the correlations relating to IJV-CSA on that side, which is exactly what was observed in the MS patients. Therefore, the fact that the correlation between NPF and left IJV-CSA was r<0.1 at all levels in the MS patients suggests that the normal biomechanical relationship between the left IJV and the CSF pulse has broken down in this group, presumably due to the rerouting of venous blood through other vessels.

Collectively, the results of the PLSCA (Fig 2 and Table 3) and the correlation analysis (Tables 4 and 5) confirm the presence of a biomechanical link between the IJVs and aqueductal CSF pulse in the healthy individuals, which although also present in the MS patients appears to be altered in this group. As such, this finding supports earlier work linking CSF pulsatility in the AoS with cerebral venous outflow in healthy subjects [6, 7]. Having said this, we were surprised to find, after controlling for BMI and age, that in both cohorts there was a general tendency for the magnitude of NNF and NPF to increase as the IJVs became larger. Given that the IJVs are thin-wall floppy vessels, which readily expand in response to any increase in blood pressure, the most likely explanation for this positive relationship is that larger CSAs are indicative of raised venous pressure in the IJVs, something that is associated with an increase in cerebral venous pressure [6]. Raised IJV pressure can occur for a variety of reasons—for example due to an increase in central venous pressure [42], or alternatively, due to constriction of the vessels down-stream of the IJV [11]. Enlarged IJVs can also occur due to increased venous blood flow. However, this too is also indicative of raised IJV pressure, since in order to ‘inflate’ the vessel walls to accommodate the additional venous flow it is necessary to increase local blood pressure. Consequently, although we cannot be certain as to why particular IJVs might enlarge, we can conclude that larger IJV-CSAs are indicative of elevated IJV pressure, and that this in turn is indicative raised cerebral venous pressure, something that has been shown to be associated with increased CSF pulsatility in the AoS [6].

While our findings highlight differences between the healthy individuals and the MS patients regarding the IJVs in the lower neck, it is important to note that we did not investigate differences between males and females in this study. In middle age, the prevalence of cardiovascular disease is significantly lower in women compared with men, and it may be that this has an influence on the vasculature of the neck. Endothelial progenitor cells, which have the capacity to form new blood vessels and contribute to vascular repair [43], have been shown to be present in higher quantities in females of reproductive age due to elevated estrogen levels, with the result that endothelial dysfunction is generally lowered in women [44]. It may be therefore, that the unique angiogenic properties of the female reproductive system, together with stem cell regulatory molecules [45] influence the structure of the IJVs. Further work, will therefore be required to investigate the impact of gender related differences on the relationship between IJV-CSA and aqueductal CSF pulsatility in both healthy individuals and MS patients. The cross-sectional design of the study also meant that it was not possible to investigate how the above relationship changed as the subjects aged, or indeed, if the observed vascular changes in any way preceeded the onset of MS. It is therefore recommended that further longitudinal studies be undertaken to investigate whether or not changes in the IJV / aqueductaral pulse relationship are in any way a precursor of neuronal damage.

In conclusion, our study provides evidence of a biomechanical link in healthy individuals between the cerebral venous outflow and the motion of the CSF pulse in the AoS, which is independent of age, BMI and cardiovascular risk factors. In healthy individuals, increased IJV-CSA at the lower cervical levels, indicative of raised IJV pressure, is linked with increased CSF pulsatility in the AoS. However, this relationship appears to be profoundly altered in MS patients, particularly on the left hand side, suggesting the presence of physiological changes associated with the cerebral venous drainage system.

## Supporting Information

S1 Table Complete dataset of the clinical, MRI and MRV data for the MS patients and healthy individuals who participated in the study. (XLS)
